# Pharmacological Treatment of Pain and Agitation in Severe Dementia and Responsiveness to Change of the Italian Mobilization–Observation–Behavior–Intensity–Dementia (I-MOBID2) Pain Scale: Study Protocol

**DOI:** 10.3390/brainsci12050573

**Published:** 2022-04-29

**Authors:** Damiana Scuteri, Marianna Contrada, Teresa Loria, Paolo Tonin, Giorgio Sandrini, Stefano Tamburin, Pierluigi Nicotera, Giacinto Bagetta, Maria Tiziana Corasaniti

**Affiliations:** 1 Pharmacotechnology Documentation and Transfer Unit, Preclinical and Translational Pharmacology, Department of Pharmacy, Health and Nutritional Sciences, University of Calabria, 87036 Rende, Italy; giacinto.bagetta@unical.it; 2 Regional Center for Serious Brain Injuries, S. Anna Institute, 88900 Crotone, Italy; mariannacontrada@gmail.com (M.C.); patonin18@gmail.com (P.T.); 3 Casa Giardino RSA, 88836 Cotronei, Italy; teresaloria@libero.it; 4 Department of Brain and Behavioral Sciences, IRCCS C. Mondino Foundation Neurologic Institute, University of Pavia, 27100 Pavia, Italy; giorgio.sandrini@unipv.it; 5 Department of Neurosciences, Biomedicine and Movement Sciences, University of Verona, 37100 Verona, Italy; stefano.tamburin@univr.it; 6 German Center for Neurodegenerative Diseases (DZNE), 53111 Bonn, Germany; pierluigi.nicotera@dzne.de; 7 Department of Health Sciences, University “Magna Graecia” of Catanzaro, 88100 Catanzaro, Italy; mtcorasa@unicz.it

**Keywords:** dementia, pain, agitation, I-MOBID2, responsiveness

## Abstract

Up to 80% of Alzheimer’s disease (AD) patients in nursing homes experiences chronic pain and 97% develops fluctuant neuropsychiatric symptoms (NPS). Agitation, associated with unrelieved pain, is managed through antipsychotics and may increase the risk of death. Evidence is accumulating in favor of analgesia for a safer, effective therapy of agitation. The Italian version of Mobilization–Observation–Behavior–Intensity–Dementia, I-MOBID2, recently validated in the Italian setting, shows: good scale content validity index (0.89), high construct validity (Spearman rank-order correlation Rho = 0.748), reliable internal consistency (Cronbach’s α coefficient = 0.751), good-excellent inter-rater (intraclass correlation coefficient, ICC = 0.778) and test-retest (ICC = 0.902) reliability, and good inter-rater and test-retest agreement (Cohen’s K = 0.744) with 5.8 min completion time. This study intends to identify the responsiveness of the I-MOBID2 based on COnsensus-based Standards for the selection of health Measurement Instruments (COSMIN) recommendations, assessing the a priori hypotheses of (1) the efficacy of painkillers administered to severe AD patients after proper pain assessment and (2) the effect of reduction of the Cohen-Mansfield Agitation Inventory (CMAI) score and of agitation rescue medications. This protocol is approved by Calabria Region Ethics Committee protocol No. 31/2017 and follows the Standard Protocol Items: Recommendations for Interventional Trials (SPIRIT) guidelines.

## 1. Introduction

Dementia represents a public health priority, with some 55 million people affected worldwide and about 41 million of them undiagnosed [1]. Among the diverse forms of dementia, Alzheimer’s disease (AD) is the most frequent standing for around two-thirds of all cases [2,3]. Despite the recent accelerated approval of aducanumab [4], without approval by the European Medicine Agency (EMA) due to contrasting efficacy results in the face of a lack of sufficient safety, disease-modifying drugs, after failures in the last years, are still not available and the spectrum of quality of life impairing disorders associated with dementia is wide. Aside from cognitive decline, which has always been considered the clinical hallmark of AD, 97% of patients presents fluctuant neuropsychiatric symptoms (NPS) during the course of the disease [5]. Moreover, the current pandemic emergency has delayed the diagnosis of NPS and of the underlying triggers, increasing the risk of mortality in these fragile patients [6,7]. Often under-recognized NPS and, in particular, depression, can represent the earliest red flag for cognitive impairment [2] and the increasing interest in clinical research in these disorders did not yield effective and safe treatment [8]. Mild Behavioral Impairment (MBI) [9,10] foreruns AD with depressive symptoms underscoring a possible phenomenon of reverse causation, according to which AD pathogenesis can induce these symptoms years before its onset [11]. Cross-sectional data obtained from 2808 patients affected by dementia referred to the European Alzheimer’s Disease Consortium demonstrated that hyperactivity, psychosis, affective symptoms, and apathy are correlated with the severity of dementia [12]. Moreover, depression, anxiety, and cognitive symptoms can forerun dementia [13]. The link between depression and Mild Cognitive Impairment (MCI) was investigated in the Cardiovascular Health Study Cognition Study, with the high cognitive function of the 2220 patients enrolled at baseline, demonstrating the higher risk to develop MCI occurring in people suffering from moderate-to-high depressive symptoms [14]. The co-occurrence of NPS before dementia development can be represented by a four-factor solution, including psychosis/apathy, depression/anxiety, irritability/persecution, and wandering/sleep problems. Depression/anxiety are present in younger patients, while psychosis is most related to cognitive deterioration [15], agitation, disinhibition, irritability, and aberrant motor behavior which increases over time with the severity of dementia [16]. NPS agitation, which is a kind of enhanced help-seeking behavior to unrelieved pain, is mostly inappropriately treated [17] in the community [18,19,20]. Agitation is identified as a form of communication in response to various sources of discomfort [21]: pain [17], depression [22], disturbance of night-time sleep pattern [23,24], constipation [25], and changes in the environment that are over or under stimulating [26]. Indeed, even the preclinical NPS profile was associated with modifications of pain perception and treatment occurring during aging [27]. In keeping with the latter, patients suffering from severe AD usually are affected by age-related comorbidities causing chronic, inflammatory, and neuropathic pain, which remains underdiagnosed due to the loss of self-report capabilities [28]. Approximately 72% of patients over 85-years is affected by chronic pain [29,30] and up to 80% of dementia patients in nursing homes experiences pain [31]. Agitation is currently treated off-label through potentially harmful neuroleptics [32], although anti-AD symptomatic drugs for the treatment of cognitive deterioration (i.e., acetylcholinesterase inhibitors and memantine) display some effectiveness [33] in delaying and preventing NPS [34,35], exacerbated by the lack of adherence [36]. Pain intensity, NPS, and the use of antipsychotics are correlated [37] and the priority of analgesia for the treatment of NPS has been demonstrated [38] since it is possible to decrease the use of antipsychotics [36,39] through appropriate, integrated pain management [40]. Therefore, pain assessment in severely uncommunicative AD patients is needed for the appropriate, efficacious, and safe therapy of pain and, consequently, NPS. To this aim, observational pain scales for uncommunicative patients affected by severe dementia are necessary. The Mobilization–Observation–Behaviour–Intensity–Dementia (MOBID2) Pain Scale is unique, taking into account the co-occurrence of musculoskeletal and visceral pain [41], with the first part assessing musculoskeletal pain, disclosing hidden pain using guided movements [42], and the second part for the detection and evaluation of pain from internal organs, head, and skin [42]. Due to its specific features and international validation as a tool with noteworthy psychometric properties, this pain scale was recently translated, cross-culturally adapted, and validated in the Italian nursing home setting in a cohort of uncommunicative AD patients over 65 years with a mini-mental state examination (MMSE) ≤ 12 [43]. The Italian Mobilization–Observation–Behaviour–Intensity–Dementia (I-MOBID2) Pain Scale proved to have a good face and scale content validity index (0.89), high construct validity (Spearman rank-order correlation Rho = 0.748), reliable internal consistency (Cronbach’s α coefficient = 0.751), good to excellent inter-rater (Intraclass correlation coefficient, ICC = 0.778) and test-retest (ICC = 0.902) reliability, and good inter-rater and test-retest agreement (Cohen’s K = 0.744) with short training and average execution time of 5.8 min [43]. In the present clinical trial protocol, the I-MOBID2 will be used with multiple aims: (1) to assess the accuracy and effectiveness of the analgesic treatments administered in patients suffering from severe dementia [44]; (2) to assess the change of agitation due to analgesic treatment measured through the Cohen-Mansfield Agitation Inventory (CMAI) [45,46] and any need for rescue medications; (3) to establish the responsiveness of the I-MOBID2, foreasmuch as, in agreement with the COnsensus-based Standards for the selection of health Measurement Instruments (COSMIN) initiative [47], the pain scale is responsive if it detects change over time in the construct to be measured, as previously established for the originally developed MOBID2 [48]. The responsiveness will be evaluated by testing the aforementioned aims 1 and 2 as *a priori* hypotheses of correlation between changes in I-MOBID2 scores and changes in other variables [48], i.e., pain treatment, CMAI score, and NPS rescue medications. 

## 2. Materials and Methods

### 2.1. Design of the Study

The protocol for the present clinical study intends to identify: (1) the efficacy of the painkillers administered to patients suffering from severe AD [44]; (2) the analgesic treatment-induced reduction of the CMAI score [45,46] and the need for rescue medications to treat agitation; (3) the responsiveness of the I-MOBID2 based on COSMIN recommendations. In particular, the *a priori* hypotheses to test are the following: (i) I-MOBID2 overall and a decrease in the first and second part pain scores after 8-weeks of analgesic treatment; (ii) where the CMAI score decreases after 8-week analgesic treatment, the duration needed for a stepwise protocol for pain treatment to reduce agitation [17] and pain-linked depression [49] in dementia, and; (iii) the need for NPS psychotropic rescue medications to be reduced after 8-weeks analgesic treatment. Psychotropic medications that are supposed to be reduced by pain treatment include neuroleptics, antidepressants, mood stabilizers, and benzodiazepines. Gabapentinoids, lamotrigine, duloxetine, and venlafaxine should be used in the treatment of neuropathic pain after proper pain diagnosis. Pain assessment will be conducted using the I-MOBID2 after a 1-week observation period and agitation evaluation through the CMAI after a 2-week observation period to become familiar with the patients. It is a psychometric tool made up of 29 items (score ranging from 29–203, with significant agitation at ≥39) rating the frequency of aggressive behavior, physical non-aggressive behavior, or verbally agitated behavior in patients suffering from dementia. The influence on pain treatment and reduced agitation treatment will be assessed through the Timed “Up and Go” (TUG) test [50], recommended for the assessment of basic functional mobility for frail elderly persons and for patients with dementia [51]. Patients able to move will be observed and timed while rising from an armchair, walking 3 m, turning, walking back, and sitting down again, after baseline assessment, rating a score corresponding to the seconds taken to complete the tasks [50]. 

### 2.2. Procedure

#### 2.2.1. I-MOBID2

As previously occurred during the I-MOBID2 validation study, the nurses will receive a 2-h training and will perform a baseline 1-week observation of the patients to familiarize themselves with them and to assess pain using the I-MOBID2. According to the instructions for the use of the tool, they will explain clearly to the patients what will happen, asking “Mrs., can you please open and close your left hand? I will help you!” [42]. For the first part consisting of items 1–5, standardized active, guided movements will be executed by the operator if the patient is not able to perform on his own. For each item, nurses will ask, “How intense do you regard the pain to be?” [42], and subsequently, rate the inferred pain intensity on the 0–10 point numeric rating scale (NRS) provided. For the assessment of the second part, the nurses will rate intensity based on pain behaviors observed on the same day or during the previous days, i.e., the baseline week of observation, as it is likely to originate from internal organs, head, and skin. According to the behavioral indicators highlighted (pain noises, facial expression, and defense), the nurses will cross/shade/circle the pain locations on the pain drawing provided to unravel the dermatomal, sclerotomal, myotomal, or combined pain distribution [52] and indicate the percentage of the body surface in pain [53,54]. A single cross involving all areas of the head and the sacroiliac joint will be computed as two marks covering both sides. At this point, each item from 6 to 10 of the I-MOBID2 will be rated as inferring pain intensity from the internal organs, head, and skin on the provided NRS. After completion of both parts, an independent overall pain intensity score will be rated using the NRS. The timeline of the study with pain assessment according to I-MOBID2 is illustrated in Table 1.

#### 2.2.2. CMAI

In agreement with the instructions manual of the CMAI, it is a caregivers’ rating questionnaire consisting of 29 agitated behaviors: each frequency of presentation will be rated on a 7-point scale based on the two weeks preceding its administration. In particular, the frequency of behavior occurrence is rated as follows: never; less than once a week; once or twice a week; several times a week; once or twice a day; several times a day; several times an hour. Since each behavior can include a wide spectrum of disorders, the raters and the respondents will be provided with a detailed description of behaviors, explaining that it is necessary to pay attention and include also similar but not exactly cited behaviors in the closest related item. In this case, the rater will be provided with appropriate training and will conduct the interview with the caregiver familiar with the patients. He will explain the importance of making this assessment and what is going to happen, providing the respondent with a copy of the scale several days before, reading aloud each item and doing the face-to-face interview without influencing him, in a quiet room, avoiding interruptions. Moreover, a rating of disruptiveness of the observed behaviors will be performed, asking for every behavior that has occurred and if it is disruptive to the staff: Not at All; A little; Moderately; Very Much; Extremely. The numeric rating scale corresponding is as follows: 1 = Never; 2 = Less than once a week but still occurring; 3 = Once or twice a week; 4 = Several times a week; 5 = Once or twice a day; 6 = Several times a day; 7 = Several times an hour. The scores will average the frequency of occurrence within the two previous weeks considered. For the I-MOBID2, about 5–6 min will be needed for completion, whereas 20 min for CMAI. The protocol for agitation assessment through the CMAI is shown in Table 2.

### 2.3. Pain Treatment

No intervention drugs out of usual care will be used. The patients meeting the inclusion criteria will be enrolled and randomly allocated to two groups: (1) usual care and (2) analgesic treatment based on the WHO analgesic ladder according to the assessment of intensity. In particular, oral non-steroidal anti-inflammatory drugs (NSAIDs) including naproxen, ibuprofen, and diclofenac will be considered for inflammatory musculoskeletal pain and celecoxib in case of chronic osteoarthrosis, after the failure of acetaminophen, only for short periods as recommended by the American Geriatric Society (AGS) panel [55,56], to reduce the gastrointestinal, renal, and cardiovascular adverse reactions [57,58,59]. In the case of warfarin concurrent use, its dose will deserve adjustment to prevent prolongation of the international normalized ratio and, thus, hemorrhage risk [60]. On the other side, for the treatment of neuropathic pain, gabapentin/pregabalin [61] will be used or serotonin-noradrenaline reuptake inhibitors (SNRIs, i.e., duloxetine, venlafaxine) [62], instead of tricyclic antidepressants (TCAs, e.g., amitriptyline) for their cardiovascular contraindications [63]. Severe chronic pain conditions, especially of a mixed nature, could require opioids, such as tramadol, tapentadol, buprenorphine, or transdermal fentanyl after effective dose titration [64,65]. For all the analgesic therapy prescriptions, the key strategy to ‘‘start low and go slow’’ will be applied [66], adjusting dosages in case of diseases associated with liver and/or renal failure. The progression will go from non-opioid analgesics such as acetaminophen to anti-inflammatory medications, drugs for neuropathic pain treatment, and finally, to opioids, according to the doses established by the AGS in 2002 [56], due to the lack of high quality/certainty evidence for the implementation of algorithm-based treatments for pain treatment in this fragile population [67]. The raters and the nurses administering the drugs will be blinded to the group allocation. Related concurrent drugs, i.e., acetylcholinesterase inhibitors and memantine, psychotropic drugs (neuroleptics, antidepressants, benzodiazepines, and mood stabilizers), anti-inflammatory and analgesic agents will be kept stable for 4 weeks before recruitment. As-need painkillers and psychotropic rescue medications will be allowed and monitored throughout the study.

**Figure 1:**
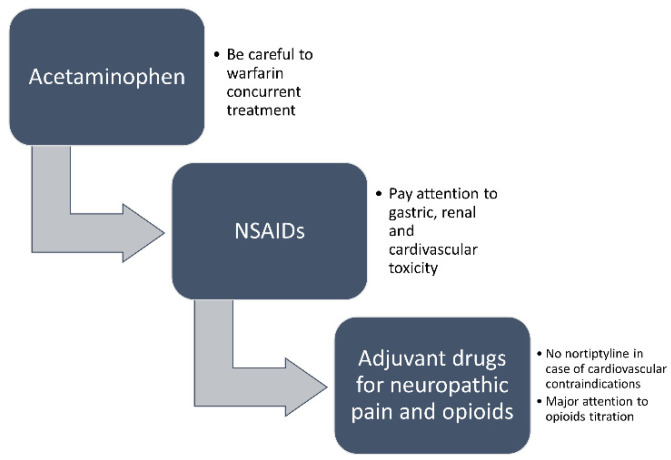
Schedule of analgesic treatment after observational pain assessment.

Figure 1 Analgesic treatment.

### 2.4. Inclusion Criteria

This is a multicentre trial involving nursing home consecutive patients. The inclusion criteria are the following: age ≥ 65 years; MMSE ≤ 12; informed consent signed by a legal representative. In particular, since GDS [68] and FAST [69] staging combined assessments exert nearly three times AD variance in the temporal course with respect to MMSE, that is the change in measure versus the change in time [70,71], AD staging for inclusion will be of GDS/FAST > 5. Patients with a diagnosis of AD based on the diagnostic and statistical manual of mental disorders (DSM)-5 will be enrolled and recruited independently on presumed pain or agitation. The assumption of needed authorized concurrent therapies for the treatment of agitation is allowed, but if treated with acetylcholinesterase inhibitors and memantine, psychotropic drugs (neuroleptics, antidepressants, benzodiazepines, and mood stabilizers), anti-inflammatory and analgesics, these will have to be kept stable for 4 weeks before recruitment to be included. The presence or history of other psychiatric disorders or neurological conditions represents the only exclusion criterion. The inclusion and exclusion criteria are reported in Table 3.

### 2.5. Ethical Approval

This study protocol follows the Standard Protocol Items: Recommendations for Interventional Trials (SPIRIT) guidelines [72,73]. This clinical trial was approved by the Ethics Committee, Section for Northern Calabria, Calabria Region, protocol No. 31/2017. According to the D.lgs 196/2003, the Helsinki agreements and subsequent amendments, the Good Clinical Practice and current legislation, the Guidelines for the treatment of personal data in clinical trials of 24 July 2008, and in accordance with European data protection legislation, each participant or his/her legal representative will be required to sign a consent form as acceptance of all aspects of the study contained in the patient information sheet and as a consequent expression of his willingness to participate in the study. The information sheet will be duly illustrated to the subjects or legal representatives by the study staff and the same staff will ensure that the consent form is properly signed and dated by all the parties involved before any procedure foreseen by the protocol is carried out. No funding was received for this trial. This is a non-profit study, in which no form of remuneration is foreseen for study participants and all the staff involved. The results of the trial will be published in an anonymous form ensuring confidentiality. A final report will be published and discussed illustrated during scientific conferences. There is no trial sponsor and data monitoring committee members are independent and do not have competing interests. 

### 2.6. Statistical Analysis

Patients are considered in pain when the I-MOBID2 items or the overall pain intensity are scored ≥ 3 and suffering from agitation with a CMAI score ≥ 39 [48]. Descriptive statistics will be used for patients’ characteristics. The correlation between the pain score reduction and established analgesic treatment will be analyzed by the paired sample *t*-test and the correlations with the CMAI and psychotropic drugs identified through Pearson’s correlation [48]. No sample power calculation is performed since the study is not interventional with new drugs. All the statistical analyses will be performed with Microsoft Office Excel 10 (Microsoft, Milan, Italy) ad SPSS-27 for Windows (IBM SPSS, Chicago, IL, USA).

## 3. Discussion

The high prevalence of pain in elderly persons in nursing homes (over 65 and with a mean of 83 years of age [74]) has been widely known and demonstrated, even before the early 2000s, in the face of under-treatment mainly due to cognitive impairment, reporting that cognitively impaired patients receive significantly fewer analgesic drugs and with reduced dosage [75]. Safe and effective therapy with analgesics in the oldest patients, in particular those subjected to cognitive impairment and post-stroke pain [76], is still poor and, they are considered inadequate for assessment, with these patients usually unjustifiably excluded from clinical trials [77]. This occurs also for migraines [78,79,80] since they rarely arise after the age of 50 years, but around 85.9% of patients over 65 years in a migraineurs sample reported episodic or chronic migraines appeared for the first time before 50 years resulting in medication overuse headache (MOH) in the 38% of cases [81]. Appropriate use of painkillers, in terms of amount, dosage, and quality, is needed for the elderly with cognitive impairment in comparison with their cognitive functioning peers. To this aim, effective and feasible pain assessment scales are unavoidable and fundamental to engineering clinical trials to establish: (1) pharmacodynamic and pharmacokinetic profiles of drugs in the fragile older population known to present physiological differences and variability in response to medications [82]; (2) information about polypharmacy [83], remarkably serious for antipsychotics and psychotropic drugs as some 49.7% of people with advanced dementia ≥ 65 years are prescribed and administered five or more medications and, in 39% of cases, at least one is a potentially inappropriate medication (PIM), based on the Beers Criteria [84], and; 3) impact of drug-to-drug [85]/herbal medicines interactions [86]. Phytocomplexes endowed with analgesic and non-benzodiazepine-like anxiolytic effects [2,87,88,89,90,91] deserve investigation for pain [92] and, consequently, agitation [36] treatment devoid of serious adverse reactions. It is an established fact that pain in 20% of the elderly is chronic [93], treated for at least 6 months [93], and unrelieved in up to 80% of cases [94]. In the considered context, i.e., the Italian setting, the Italian Silver Network Home Care project illustrated that about 49% of patients suffer from daily pain and only 25% of them receives a WHO I level analgesic [95]. In the frame of this complex scenario, the purpose of the present clinical trial is to shed light on the correlation between appropriate pain treatment and the reduction of agitation and, consequently, of psychotropic drugs. This would also prove the I-MOBID2 responsiveness to change in agreement with the COSMIN panel. Since pain treatment also reduces depression and a wide spectrum of behavioral disorders in dementia, the impact of appropriate pain pharmacological treatment on the reduction of depression and psychological symptoms will be investigated in further clinical trials.

## Figures and Tables

**Table 1:** Timeline of pain assessment.

		STUDY PERIOD									
	Enrolment	Allocation	Allocation	Post-Allocation:Interventions							
TIMEPOINT	*-within* *2 weeks*	Behavioral Baseline Assessment *1 week*	Baseline *1 week*	*week1*	*week2*	*week3*	*week4*	*week5*	*week6*	*week7*	*week8*
**ENROLMENT:**	X										
**Eligibility screen**	X										
**Informed consent**	X										
***Physical examination***	X										
**Allocation**			X								
**INTERVENTIONS:**											
***Analgesic treatment upon assessment***				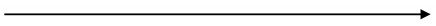							
***Usual treatment***				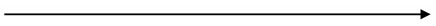							
**ASSESSMENTS:**											
***I-MOBID-2***			baseline observation	X	X	X	X	X	X	X	X
***Timed “Up and Go” (TUG)***			baseline observation	X	X	X	X	X	X	X	X

Schedule of enrollment, interventions, and pain assessment through the Italian Mobilization–Observation–Behaviour–Intensity–Dementia (I-MOBID2) Pain Scale based on the Standard Protocol Items: Recommendations for Interventional Trials (SPIRIT) guidelines.

**Table 2:** Timeline of agitation assessment.

	STUDY PERIOD									
	Enrolment	Allocation	Post-Allocation:Interventions							
TIMEPOINT	*-within* *2 weeks*	*Baseline* *2 weeks*	*week1*	*week2*	*week3*	*week4*	*week5*	*week6*	*week7*	*week8*
**ENROLMENT:**	X									
**Eligibility screen**	X									
**Informed consent**	X									
***Physical examination***	X									
**Allocation**		X								
**INTERVENTIONS:**										
***Analgesic treatment upon assessment***			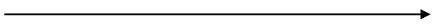							
***Usual treatment***			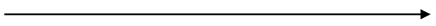							
**ASSESSMENTS:**										
***CMAI***		Baseline observation	X	X	X	X	X	X	X	X

Schedule of enrollment, interventions, and agitation assessment through the Cohen-Mansfield Agitation Inventory (CMAI), according to the Standard Protocol Items: Recommendations for Interventional Trials (SPIRIT) guidelines.

**Table 3:** Inclusion/exclusion criteria.

Inclusion Criteria	Exclusion Criteria
Males or females ≥ 65 years of age; DSM-5 criteria for AD; MMSE ≤ 12 GDS/FAST > 5 Related concurrent drugs, i.e., acetylcholinesterase inhibitors and memantine, psychotropic drugs (neuroleptics, antidepressants, benzodiazepines, and mood stabilizers), anti-inflammatory and analgesic agents kept stable for 4 weeks before recruitment; Informed consent signed by a legal representative.	Presence or history of concurrent or previous psychiatric disorders or neurological conditions (i.e., epilepsy and schizophrenic disorders).

Criteria for eligibility for the clinical study.

## Data Availability

Not applicable.
